# Osteochondral lesions in children with juvenile idiopathic arthritis

**DOI:** 10.1186/1546-0096-11-18

**Published:** 2013-05-01

**Authors:** Liisa Kröger, Eija Piippo-Savolainen, Erja Tyrväinen, Pekko Penttilä, Heikki Kröger

**Affiliations:** 1 Department of Paediatrics, Kuopio University Hospital, Kuopio FIN-70211, Finland; 2 Department of Radiology, Kuopio University Hospital, Kuopio, Finland; 3 Department of Orthopaedics, Traumatology and Hand Surgery, Kuopio University Hospital, Kuopio, Finland; 4 Bone and Cartilage Research Unit, University of Eastern Finland, Kuopio Campus, Finland

**Keywords:** Juvenile arthritis, JIA, Osteochondritis, Joint, Pain

## Abstract

**Background:**

Joint pain and swelling are typical symptoms in children with juvenile idiopathic arthritis (JIA) and these are often related to inflammation of the joint. Juvenile osteochondritis dissecans (JOCD), that is separation of a bone-cartilage segment from the articular surface, can manifest with similar symptoms.

**Findings:**

We studied thirteen cases of osteochondritis dissecans lesions (OCD) in children with JIA. There were nine girls and four boys with a mean age of 6.5 (2–12) years at the time of diagnosis of JIA. Mean time between diagnosis of JIA and manifestation of OCD was 5.5 (1–11) years. Indications for MRI were the presence of pain or discomfort in the joint, despite otherwise effective treatment, with no evidence from ultrasound examination of any obvious signs of active inflammation. The most common location of osteochondral lesion was the knee, although the ankle joint was affected in one case. Five patients had lesions in both knees. Operative treatment was needed in eight cases (joints).

**Conclusions:**

Pain, and minor dysfunction of the joint are common complaints of children suffering from JIA. Earlier research has discounted the possibility of children who were not athletes presenting with this condition. However, this study demonstrates that these lesions also seem to be relatively common in patients with JIA. When there is no sign of inflammation, the possibility of OCD must therefore be considered in these children.

## Findings

### Introduction

Osteochondritis dissecans (OCD) refers to the separation of a subchondral bone segment from the articular surface. The aetiology of juvenile osteochondritis dissecans is not known but it is suspected that it is related to endocrine disorders, epiphyseal or vascular abnormalities, or repetitive trauma. OCD is found more frequently in children involved in organized sports activities Symptoms may vary from locking or clicking to pain and swelling of the joint. The natural history in children can be self-limiting and many lesions can heal without ever being diagnosed [1-3].

Juvenile idiopathic arthritis (JIA) is defined as arthritis of unknown aetiology which starts before the age of 16 years and persists for more than six weeks. JIA is the most common chronic rheumatic disease of childhood, with a reported prevalence of between 16-150/ 100 000 in high-income countries. Juvenile arthritis is classified according to seven different categories based on clinical features (i.e. the number of joints inflamed), family history and laboratory markers Thus the diagnosis of JIA consists of a heterogenous group of chronic inflammatory arthritis [4]. The clinical care of children with JIA has improved significantly over the past decade. First-line treatment consists of nonsteroidal anti-inflammatory analgesics (NSAIDs) and intra-articular steroid injections often followed by methotrexate and, in resistant cases, by biologic therapy [4].

Previously, conventional radiography has been the mainstay of imaging in JIA. However, current practice is to use ultrasound for diagnosis and Magnetic Resonance Imaging (MRI) and ultrasound for monitoring disease activity, progression and response to treatment [5]. When trying to determine the cause of pain and discomfort in the joints of children with JIA, we found osteochondral lesions to be relatively common.

### Cases

The average number of JIA patients visiting our paediatric rheumatology clinic varies annually between 170 and 230. The annual number of new JIA cases varies between 15–25. During 2007–10, osteochondral lesions were found in thirteen patients with a previous diagnosis of JIA. There were four boys and nine girls. Twelve patients had lesions in the knee (five of these had lesions in both knees); in one patient the affected joint was the ankle, with the lesion being in the talus. Therefore the total number of affected joints was 17 knees and one ankle (Table 1).

**Table 1:** Clinical characteristics of the patients with osteochondral (OCD) lesions

**Patient**	**JIA type**	**Sex, age at diagnosis**	**Medication**	**Age at diagnosis of OCD**	**Number of Ia. injections**	**OCD location, acc to MRI**	**Operative treatment**
1	Extended polyarthritis	M, 4	MTX Etanercept Infliximab Abatacept	10	12	Femur	-
						lateral	
2	Polyarthritis	F, 10	MTX	12	3	Femur lateral	+
				15	7	(dx)	
						Femur,	
						lateral (sin)	+
3	Oligoarthritis	M, 12	MTX	15	4	Femur lateral	+
4	Extended Polyarthritis	M, 5	MTX	10	9	Femur lateral (l.a.)	-
5	Oligoarthritis	F, 10	MTX	13	1	Femur lateral	+
6	Polyarthritis	M, 11	MTX	12	5	Femur medial and lateral l.a.	-
7	Oligoarthritis	F, 2	MTX	10	2	Femur medial and lateral	+
8	Extended polyarthritis	F, 3	MTX Oxichlorin Infliximab Adalimumab	14	3	Femur medial	-
9	Polyarthritis	F, 2	Leflunomide	11	9	Talus	+
10	Extended polyarthritis	F, 2	MTX, SSZ Leflunomide Etanercept Infliximab	10	12	Femur medial	-
11	Polyarthritis	F, 10	MTX	14	3	Femur medial l.a.	+
12	Polyarthritis	F, 9	MTX	10	3	Femur (dx) medial and lateral	-
						Femur medial (sin)	
13	Polyarthritis	F, 4	MTX Leflunomide Etanercept	15	5	Femur medial	+

Abbreviations: Ia. = intra-articular, MTX = methotrexate, SSZ = sulphasalazine, M = male, F = female.

The indication for imaging was the presence of pain or discomfort in the joint, despite otherwise effective treatment for inflammation. The patients mainly complained of pain, swelling or locking of the joint. Where ultrasound showed no indication of inflammation, examination was continued either with conventional plain radiography (in eight cases) or MRI with contrast media. In four of these eight cases the plain radiograph also failed to demonstrate any abnormality, and examination continued using MRI. In addition, where abnormality was found in plain radiography or where assessment of the activity of the disease was the main indication of imaging, MRI was then considered appropriate.

The mean age of patients at the time of diagnosis of JIA was 6.5 years (range 2–12 years). Six of the patients suffered from rheumatoid factor negative polyarthritis, three from oligoarthritis and four were diagnosed with extended polyarthritis (Table 1). Antinuclear antibodies were found in three cases. Four patients reported recent minor trauma of the joint.

All the children were under the care of paediatric rheumatologists who supervised their drug therapy and follow-up. All were treated with methotrexate during at least some period of the disease. In three cases, because of adverse effects, methotrexate was replaced by leflunomide. Four patients were diagnosed with extended polyarthritis; in these cases the disease was controlled not by conventional treatment but by biologics, as is indicated for this category. All four were first given TNFα modulators (etanercept, infliximab or adalimumab); in one case this was later replaced by abatacept (Table 1). In addition, intra-articular glucocorticoid injections were given where needed. The average number of intra-articular injections given in the knee or ankle joint was 1.3 / year and the number of injections was highest in patients who later needed biologics (Table 1). Intra-articular injections were also given more frequently in the early phase of the disease when inflammation was not controlled by antirheumatic drugs. The mean time between diagnosis of JIA and discovery of OCD lesions was 5.5 years (1–11 years).

Five patients had osteochondral lesions in both knees, four patients on both knees simultaneously (patients 4, 6, 11 and 12), one with an interval of three years (patient 2). Lesions were found in the lateral condyle in nine knees (six patients); only the medial condyle was affected in five knees (four patients). One patient (patient 6) had lesions in both condyles and both patellas were also affected. There were also two sibling pairs (patients 2 and 3, patients 4 and 8).

The number of patients needing operative treatment was 7/ 12 (58%). Indications for operative intervention were instability of the lesion or locking of the knee.

### Discussion

**Figure 1:**
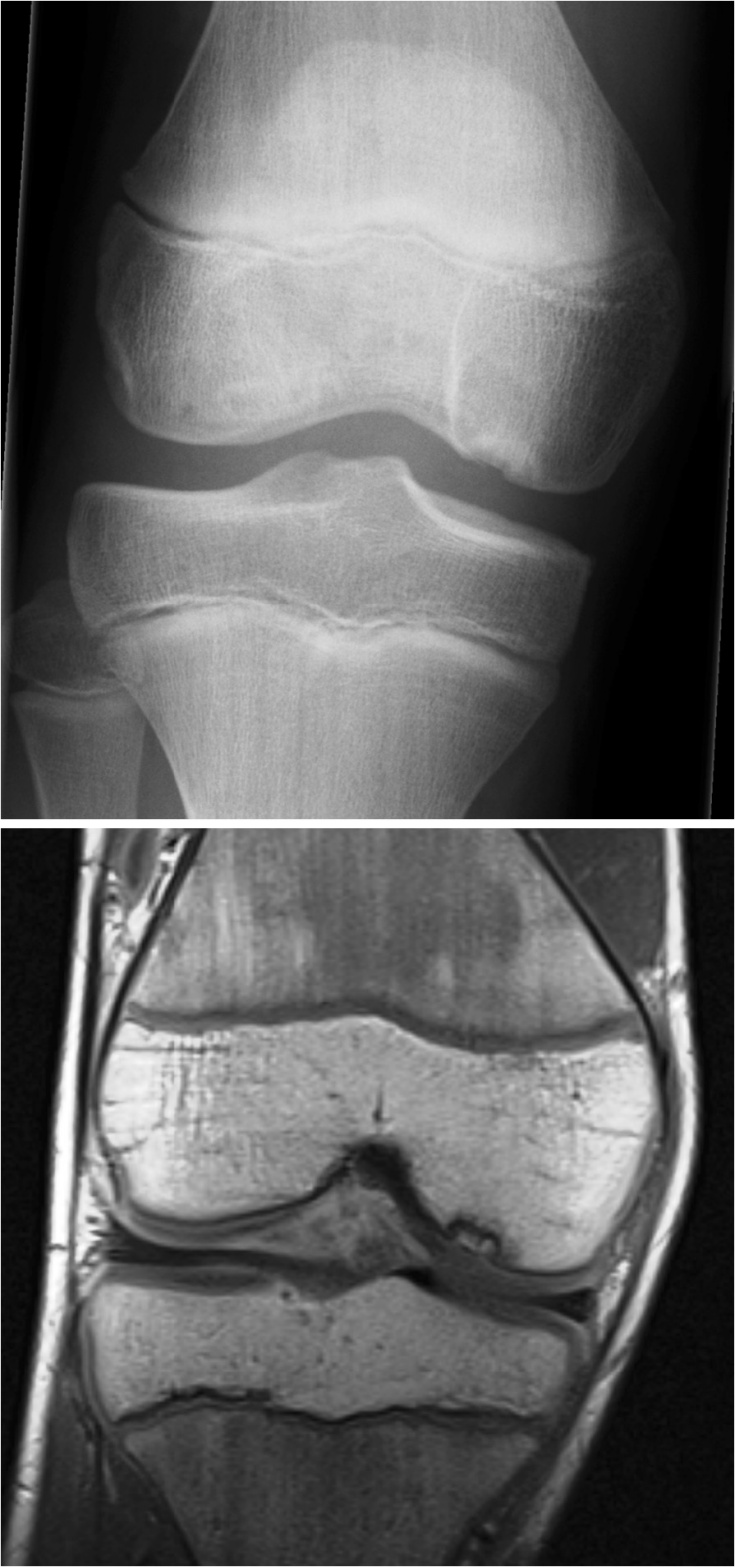
Radiograph and MRI showing an osteochondral lesion of medial femoral condyle.

Osteochondritis dissecans (OCD) is a lesion of subchondral bone, with or without articular cartilage involvement, resulting in chondral or osteochondral fragment separation (Figure 1). It is twice as common in males as in females, but the true incidence of this condition in children is not known [3,6]. In this study, there were nine females and four males. Except in cases of enthesitis-related arthritis, the prevalence of JIA is higher in females [4].

The aetiology of OCD is not yet conclusively understood, but trauma, ischemia and genetic factors have been proposed [3,6]. In this small group of patients there were two sibling pairs, suggesting a genetic cause as previously postulated [7,8]. Four patients reported minor trauma before the appearance of symptoms.

Despite the fact that this group of patients represents a wide range of severity of juvenile arthritis, the disease was relatively mild in the majority of them. The goal of treatment in JIA is to obtain total suppression of joint inflammation, and the clinical care of children with JIA has improved significantly in this respect over the past decade. First-line treatment consists of NSAIDs and intra-articular steroid injections, commonly followed by methotrexate. An important step has been understanding the mechanisms of inflammation and the development of biologic treatment [4,9]; four patients were first given TNFα-modulators and one later needed other biologics.

In addition to systemic medication, these patients also required intra-articular injections. Although the number of injections was relatively high in some cases (eg.12 injections in the same joint), these were given over a long time span. Some patients needed injections only in the early phase of the disease. It is possible that glucocorticoid injection can affect cartilage metabolism and may even increase the risk of development of OCD [10]. However, persistent synovitis is also associated with an increased risk of damage to cartilage and may thus increase the risk of development of OCD as well [4,5].

This report shows that osteochondral lesions are frequently found in patients suffering from juvenile arthritis. If the patient complains of pain , locking or swelling of the knee, imaging is indicated. In some cases it is possible to confirm or exclude the diagnosis of OCD with a plain radiograph. However, in this study, plain radiograph failed to show any abnormality in half of the cases. MRI was then used either to identify or confirm the findings. MRI may also help to evaluate the stability of the lesion and thus give information for follow-up and treatment [11,12]. It is possible that the routine use of MRI makes the prevalence of asymptomatic OCD higher [12].

In the knee joint, osteochondritis dissecans is commonly reported to involve the medial femoral condyle (85% of cases) and to be less common in inferolateral (13%) or anterior lateral femoral condyle (2%) [6]. In this study, the lateral condyle was affected in six patients, medial in four and both in three patients. The position of the lesions is therefore different from that shown in previously published material.

Treatment of juvenile OCD is dependent upon the size, location and stability of the fragment. The optimal treatment of juvenile osteochondritis is not known [13,14]. There is a possibility of spontaneous healing, but it seems that lesions with an increased size and mechanical symptoms are less likely to be healed spontaneously [13]. In this group of patients, continuous pain, locking or intra-articular loose fragments were indications for operative treatment.

### Conclusion

Pain, swelling and minor dysfunction of the joint are common complaints in children suffering from JIA. In cases with continuous pain and discomfort and no signs of inflammation, the possibility of OCD must be considered.

### Consent

Written informed consent was obtained from the patient’s guardian/parent/next in keen for publication of this report and any accompanying images.

## Abbreviations

JIA: Juvenile idiopathic arthritis; MTX: Methotrexate; SSZ: Sulphasalazine; OCD: Osteochodritis; Ia: Intra-articular

## Competing interests

None of the authors have any conflict of interest to report.

## Authors’ contributions

All authors work in Kuopio University Hospital and have conferred on the treatment of these patients. All authors have contributed to drafting and editing of the manuscript. They have read and approved the final manuscript.
